# Perturbations of Metabolomic Profiling of Spleen From Rats Infected With *Clonorchis sinensis* Determined by LC-MS/MS Method

**DOI:** 10.3389/fmolb.2020.561641

**Published:** 2020-10-06

**Authors:** Xiaoli Zhang, Xinyi Hu, Rui Chen, Beibei Sun, Yannan Gao, Shanshan Duan, Liyan Liu, Su Han

**Affiliations:** ^1^Department of Parasitology, Harbin Medical University, Harbin, China; ^2^Department of Orthopaedic Surgery, The Fourth Affiliated Hospital of Harbin Medical University, Harbin, China; ^3^Department of Nutrition and Food Hygiene, School of Public Health, Harbin Medical University, Harbin, China

**Keywords:** *Clonorchis sinensis*, infection, spleen, non-targeted metabolomics, metabolic pathway

## Abstract

Clonorchiasis is an important zoonotic parasitic disease worldwide. In view of the fact that parasite infection affects host metabolism, and there is an intricate relationship between metabolism and immunity. Metabolic analysis of the spleen could be helpful for understanding the pathophysiological mechanisms in clonorchiasis. A non-targeted ultra high performance liquid tandem chromatography quadrupole time of flight mass spectrometry (UHPLC-QTOF MS) approach was employed to investigate the metabolic profiles of spleen in rats at 4 and 8 weeks post infection with *Clonorchis sinensis* (*C. sinensis*). Then a targeted ultra-high performance liquid chromatography multiple reaction monitoring mass spectrometry (UHPLC-MRM-MS/MS) approach was used to further quantify amino acid metabolism. Multivariate data analysis methods, such as principal components analysis and orthogonal partial least squares discriminant analysis, were used to identify differential metabolites. Finally, a total of 396 and 242 significant differential metabolites were identified in ESI+ and ESI− modes, respectively. These metabolites included amino acids, nucleotides, carboxylic acids, lipids and carbohydrates. There were 38 significantly different metabolites shared in the two infected groups compared with the control group through the Venn diagram. The metabolic pathways analysis revealed that pyrimidine metabolism, aminoacyl-tRNA biosynthesis, purine metabolism and phenylalanine, tyrosine and tryptophan biosynthesis were significantly enriched in differential metabolites, which was speculated to be related to the disease progression of clonorchiasis. Furthermore, 15 amino acids screened using untargeted profiling can be accurately quantified and identifed by targeted metabolomics during clonrochiasis. These results preliminarily revealed the perturbations of spleen metabolism in clonorchiasis. Meanwhile, this present study supplied new insights into the molecular mechanisms of host-parasite interactions.

## Introduction

Clonorchiasis caused by *Clonorchis sinensis* (*C. sinensis*) is an important public health problem globally. *C. sinensis* infected at least 15 million people in countries such as China, Vietnam, South Korea, and the Russian Far East (Kim et al., 2016). Eating uncooked freshwater fish containing *C. sinensis* metacercariae is the cause of the infection. The adults of *C. sinensis* parasitize in the intrahepatic bile duct. Infection with *C. sinensis* often leads to chronic hepatobiliary diseases, such as hepatic fibrosis and cholangiocarcinoma. Notably, *C. sinensis* has been classified as a Class I carcinogen by the International Agency for Research on Cancer (Grosse et al., 2009). Our previous research had found that *C. sinensis* infection induced the dysregulation of hepatic microRNA and hepatic apoptosis in rat models (Zhang et al., 2008; Han et al., 2016). However, the molecular pathogenesis of clonorchiasis is still not completely understood.

Compared with transcriptome and proteome analyses, metabolomics measures small molecules in biological samples, which could describe the metabolic phenotype in detail and the relevant metabolic disorders (Murphy, 2020). Metabolomics has been successfully applied to several fields, such as disease diagnosis, biomarker screening, and nutrition research (Kafsack and Llinás, 2010; Khamis et al., 2017). Metabolomics provides globally dynamic changes and contributes to reveal the underlying molecular mechanisms in the diseases (Shinde et al., 2017).

Recently, more and more researchers have combined metabolomics and immunology for scientific research. It is reported that inflammation, disruption of the tricarboxylic acid (TCA) cycle, amino acids metabolism, protein synthesis and oxidative phosphorylation are all related to the immune response during infection (Nguyen et al., 2018). While, immune cells could use pentose phosphate shunt, glutamine breakdown and fatty acid oxidation to meet their metabolic and functional needs (Ganeshan and Chawla, 2014). Interestingly, interaction between immunity and metabolism also plays an important role in the pathogenesis of parasitic diseases. For example, *Toxoplasma gondii* caused metabolic recombination of host cells (Zhou et al., 2016), and the anti-inflammatory and immunoregulatory effects of steroid hormones can affect the host immune responses to infection (Chen X. Q. et al., 2017). The proliferation of B cells in primary lymphoid follicles required amino acids and lipid components to form new cell membranes and organelles in *Fasciola hepatica* infection (Saric et al., 2010). These studies show that metabolites can significantly affect the immune system, and immune inflammatory responses are also related to metabolic changes.

Given the complex relationship between immunity and metabolism, and the importance of the spleen in the immune response, the metabolic profiles of spleen in *C. sinensis*-infected rats were investigated by non-targeted ultra high performance liquid tandem chromatography quadrupole time of flight mass spectrometry (UHPLC-QTOF MS) in this study. Based on the results of untargeted profiling, amino acid metabolism was chosen for further quantifying by a targeted ultra high performance liquid chromatography multiple reaction monitoring mass spectrometry (UHPLC-MRM-MS/MS) approach. The exploration of metabolic disorders and related biochemical pathways could be useful for enhancing our understanding of the pathophysiological mechanisms in clonorchiasis.

## Materials and Methods

### Ethical Approval

This study was reviewed and ethically approved by the Medical Ethics Review Committee of Harbin Medical University. All animal experiments were performed on the basis of the Guide for the Care and Use of Laboratory Animals published by the Ministry of Science and Technology of the People’s Republic of China. In the study, we made significant efforts to reduce animal suffering and the number of animals.

### Animal Infection

Metacercariae of *C. sinensis* were collected from *Pseudorasbora parva* originating from the Songhuajiang River of Heilongjiang Province. The collection and preparation of metacercariae are described as following. First, we put the fish in an ice box at 0°C and shipped to the laboratory. Second, the fish were washed with tap water, broken up in a Waring Blender, and digested with a pepsin-HCl (0.6%) artificial gastric juice at 37°C for 12 h. Finally, the digested mixture was passed through three sieves with mesh sizes of 1000, 300, and 106 μm in sequence. A large number of pure metacercariae were harvested by centrifugation and stored at 0.1 M phosphate-buffered saline (PBS, pH = 7.4) at 4°C until used. Male wistar rats (5–6 weeks old) were purchased from the Harbin Medical University Laboratory Animal Center. The rats were fed standard laboratory food and drinking water. A total of 20 rats were individually infected orally with 50 metacercariae. Control rats (*n* = 10) were fed with 50 μl of sterile normal solution.

### Tissue Collection and Detection of Infection

*Clonorchis sinensis* undergoes rapid development and the adults develop matured at 4 weeks post infection (wpi) after infected by metacercariae (Lun et al., 2005). Our previous study also found that the hepatocyte apoptosis index of *C. sinensis*-infected rats with increased from 4 wpi, and reached a peak at 8 wpi (Zhang et al., 2008). Additionally, liver iron deposits were also found apparently at 8 wpi (Han et al., 2017). Furthermore, the same time points were also selected in other *C. sinensis* infection study for pathogenic mechanism (Lee et al., 1987; Uddin et al., 2012). Hence, at 4 and 8 wpi, rats were sacrificed and spleens were rinsed with saline solution (0.9% NaCl w/v), and stored at −80°C until analysis. In addition, control animals were sacrificed at both time points. Feces were collected weekly and microscopically examined by the Kato-Katz method to determine whether the rats had successfully infected with *C. sinensis* (Hong et al., 2003).

### Chemicals

Acetonitrile was purchased from Merck. In addition, ammonium acetate (NH_4_AC), ammonium hydroxide (NH_4_OH), formic acid (FA), and ammonium fluoride (NH_4_F) were obtained from Sigma Aldrich.

### Metabolites Extraction

A total of 50 mg of spleen sample was taken and placed in a EP tube, then added 1000 μL extraction solvent containing an internal target (V methanol: V acetonitrile: V water = 2:2:1, containing internal standard, 2-Chloro-L-phenylalanine, 2 μg/mL). Samples were homogenized in ball mill for 4 min at 45 Hz, then ultrasound treated for 5 min (incubated in ice water). After homogenization for 3 times and incubation for 1 h at −20°C to precipitate proteins, samples were centrifuged at 12,000 rpm for 15 min at 4°C. Supernatant (825 μL) was transferred into EP tubes. Extracts were dried in a vacuum concentrator without heating, and 200 μL extraction solvent (V acetonitrile: V water = 1:1) reconstitution was added into dried metabolites. Samples were vortexed for 30 s, sonicated for 10 min (4°C water bath) and centrifuged for 15 min at 12,000 rpm, 4°C. Subsequently, the clear supernatant was transferred into a fresh 2 mL LC/MS glass vial for analysis.

### UHPLC-QTOF MS Analysis

LC-MS/MS analyses were performed using an UHPLC system (1290, Agilent Technologies) with a UPLC BEH Amide column (1.7 μm 2.1 × 100 mm, Waters) coupled to TripleTOF 6600 (Q-TOF, AB Sciex) in Shanghai Biotree biotech Co., Ltd.

The mobile phase consisted of 25 mM NH_4_AC and 25 mM NH_4_OH in water (pH = 9.75) (A) and acetonitrile (B) was carried with elution gradient as follows: 0 min, 95% B; 0.5 min, 95% B; 7 min, 65% B; 8 min, 40% B; 9 min, 40% B; 9.1 min, 95% B; 12 min, 95% B, delivered at 0.5 mL min^–1^. The injection volume were 1 μL. The Triple TOF mass spectrometer was used for its ability to acquire MS/MS spectra on an information-dependent basis (IDA) during an LC/MS experiment. In this mode, the acquisition software (Analyst TF 1.7, AB Sciex) continuously evaluates the full scan survey MS data as it collects and triggers the acquisition of MS/MS spectra depending on preselected criteria. In each cycle, 12 precursor ions whose intensity greater than 100 were chosen for fragmentation at collision energy (CE) of 30 V (15 MS/MS events with product ion accumulation time of 50 ms each). ESI source conditions were set as following: Ion source gas 1 as 60 Psi, Ion source gas 2 as 60 Psi, Curtain gas as 35 Psi, source temperature 600°C, Ion Spray Voltage Floating (ISVF) 5000 or −4000 V in positive or negative modes, respectively.

During mass spectra collection, samples were placed in automatic sampler at 4°C. To monitor the stability and repeatability of the analytical system, quality control (QC) samples were prepared by pooling 10 μL of each sample and injected prior to analysis. And then blank and QC samples were injected every five samples injections throughout the analytical run.

### UHPLC-MRM-MS/MS Analysis

The targeted UPHLC-MRM-MS/MS approach was applied to verified amino acids involved in clonorchiasis. Among 4, 8 wpi and control groups, eight randomly selected rats from each group were used for investigation. An aliquot of each individual spleen sample was precisely weighed and then transferred to an Eppendorf tube. After the addition of two little steel balls and 1000 μL of extract solvent (precooled at −20°C acetonitrile-methanol-water, 2:2:1, containing isotopically labeled internal standard mixture), the samples were vortexed for 30 s, homogenized at 40 Hz for 4 min, and sonicated for 5 min in ice-water bath, repeated for three times. Next, the samples were incubated at −40°C for 1 h, followed by centrifugation for 15 min at 12,000 rpm, 4°C. After that, 80 μL supernatant was transferred to an auto-sampler vial for UHPLC-MS/MS analysis.

The LC-MS/MS analyses were also performed in Shanghai Biotree biotech Co., Ltd. The mobile phase A was 1% formic acid in water, and the mobile phase B was 1% formic acid in acetonitrile. The column temperature was set at 35°C. The auto-sampler temperature was set at 4°C and the injection volume was 1 μL. An Agilent 6460 triple quadrupole mass spectrometer (Agilent Technologies), equipped with an AJS electrospray ionization (AJS-ESI) interface, was used for assay development. Typical ion source parameters were: capillary voltage = +4000/−3500 V, Nozzle Voltage = +500/−500 V, gas (N2) temperature = 300°C, gas (N2) flow = 5 L/min, sheath gas (N2) temperature = 250°C, sheath gas flow = 11 L/min, nebulizer = 45 psi. Agilent MassHunter Work Station Software (B.08.00, Agilent Technologies) was employed for MRM data acquisition and processing.

### Data Preprocessing and Annotation

MS raw data files (.wiff) were converted to the mzXML format using ProteoWizard, and processed by R package XCMS (version 3.2). The preprocessing results generated a data matrix that consisted of the retention time (RT), massto-charge ratio (*m*/*z*) values, and peak intensity. R package CAMERA was used for peak annotation after XCMS data processing. Compound identification of metabolites was performed by comparing the accuracy of *m*/*z* values (<25 ppm), and MS/MS spectra were interpreted with an in house MS2 database (Shanghai Biotree biotech Co., Ltd.) established with authentic standards.

### Statistical Analysis

After preprocessing the raw data, multivariate statistical analysis (principal component analysis, PCA; orthogonal partial least squares discriminant analysis, OPLS-DA) was performed using SIMCA software (V14.1, Sartorius Stedim Data Analytics AB, Umea, Sweden). The parameters values of R2 and Q2 were verified the fitness and predictive ability of the model. And the OPLS-DA permutation test proves that the original model has excellent stability and there is no over-fitting phenomenon. The *P*-value of Student’s *t*-test was less than 0.05, and the Variable Importance in the Projection (VIP) of OPLS-DA model was greater than 1, so as to identify the metabolites expressed differently. Log2-fold change (FC) based on metabolite abundance was used to assess the variation of the metabolites. The box-plots (data range, quartile range, and median values) were used to illustrate the spread and differences of samples from the target analysis.

Heatmaps were applied to describe the unbalanced metabolic profiles among *C. sinensis*-infected and control rats. Euclidean distance algorithm for similarity measure and average linkage clustering algorithm (clustering uses the centroids of the observations) for clustering were selected when performing hierarchical clustering. Based on deferentially expressed metabolite data (log2-scaled), heatmaps were structured by the MultiExperiment Viewer (MeV) v. 4.9 software^1^.

Metabolites were identified by comparing the molecular mass data (*m*/*z*) of samples with the KEGG^2^ database. According to online Kyoto Encyclopedia of Genes and Genomes (KEGG) database^3^, we retrieved metabolites and extracted the corresponding pathways in KEGG. Compared with controls, further screening was performed on the pathway affected by *C. sinensis* infection using MetaboAnalyst 3.0^4^.

## Results

### Metabolic Profiles of Spleen During *C. sinensis* Infection

The spleen metabolites in *C. sinensis*-infected rats was analyzed by the non-targeted UHPLC-QTOF MS system. There were 2270 and 2282 ions detected in ESI+ and ESI− mode, respectively. A series of preparations and collation of the original data were performed, in order to better analyze the data. After deleting low-quality ions [relative standard deviation (RSD) >30%], a total of 2243 and 2274 ions in samples were recognized in ESI+ and ESI− mode, respectively.

**FIGURE 1:**
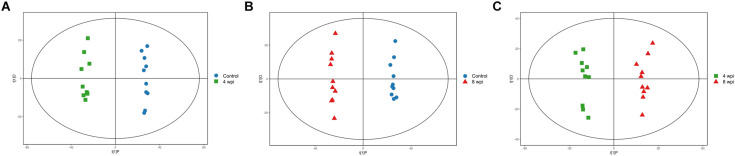
Orthogonal partial least squares discriminant analysis (OPLS-DA) score scatter plots of splenic metabolites during *C. sinensis* infection in ESI+ mode. **(A)** OPLS-DA score scatter plot of 4 wpi vs control in ESI+ mode; **(B)** OPLS-DA score scatter plot of 8 wpi vs control in ESI+ mode; **(C)** OPLS-DA score scatter plot of 8 vs 4 wpi in ESI+ mode. Control, healthy control; 4 wpi, 4 weeks post infection; 8 wpi, 8 weeks post infection.

**FIGURE 2:**
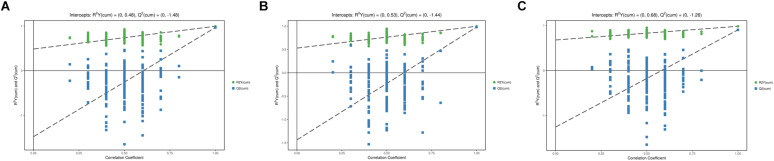
Permutation test of OPLS-DA model showing the stability of the model in ESI+ mode. **(A)** Permutation test of OPLS-DA model of 4 wpi vs control in ESI+ mode; **(B)** Permutation test of OPLS-DA model of 8 wpi vs control in ESI+ mode; **(C)** Permutation test of OPLS-DA model of 8 vs 4 wpi in ESI+ mode. The abscissa indicates the displacement retention of the permutation test, and the ordinate indicates the value of R2Y or Q2. The green dot indicates the R2Y value obtained by the displacement test, the blue square indicates the Q2 value obtained by the permutation test, and the two dotted lines indicate the regression lines of R2Y and Q2, respectively. The point where the displacement retention is 1 is R2Y and Q2 of the original model.

In the PCA model, QC samples were successfully separated from the tested samples and clustered together. It was identified that the UHPLC-QTOF MS analysis obtained better stability and reproducibility. However, the PCA score plots could not clearly distinguish the infected group from the control group (Supplementary Figure S1). The parameters R2 and Q2 confirmed the validity of the PCA model as follows: ESI+ mode, R2X = 0.58; ESI− mode, R2X = 0.58 (Supplementary Table S1). Therefore, the OPLS-DA was used to analyze the metabolites and relationships among different infection groups. The OPLS-DA score scatter plots showed that there were significant separation between the different infection groups and the control group in the ESI+ and ESI− modes, respectively (Figure 1 and Supplementary Figure S2). According to the permutation test results, the OPLS-DA model was proved to have good robustness without over fitting (Figure 2 and Supplementary Figure S3). In addition, the OPLS-DA model parameter are shown in Supplementary Table S1.

### Differential Metabolites During Different Infection Periods

**FIGURE 3:**
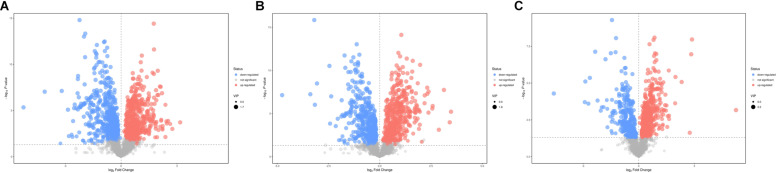
Volcano plot representation of the differential metabolites identified in ESI+ mode. **(A)** 4 wpi vs control; **(B)** 8 wpi vs control; **(C)** 8 vs 4 wpi. Each point in the map represents a metabolite. The size of the scatter represents the VIP value of the OPLS-DA model, and the larger the scatter, the larger the VIP value. Scatter color represents the final screening result, red represents significant up-regulation, blue represents significant down-regulation, and gray represents non-significant difference metabolites.

**FIGURE 4:**
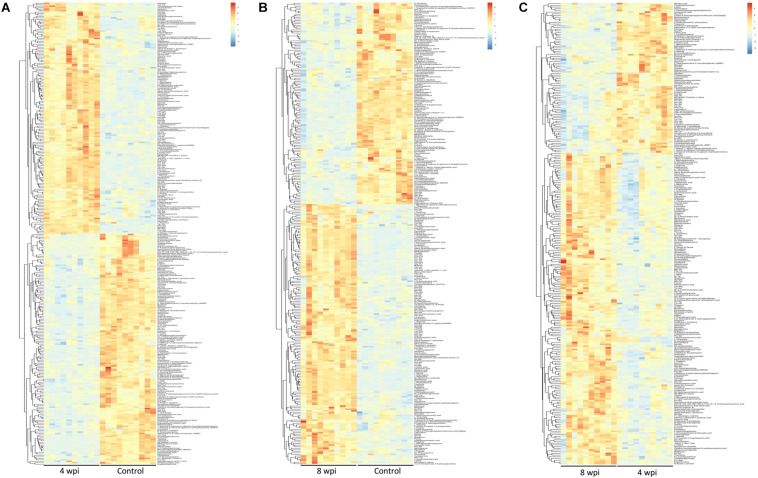
Heatmaps representation of the differential metabolites identified between different infection group vs control group in ESI+ mode. **(A)** 4 wpi vs control; **(B)** 8 wpi vs control; **(C)** 8 vs 4 wpi. The color blocks at different positions represent the relative expression of metabolites at corresponding positions, red represents up-regulated, and blue represents down-regulated.

**FIGURE 5:**
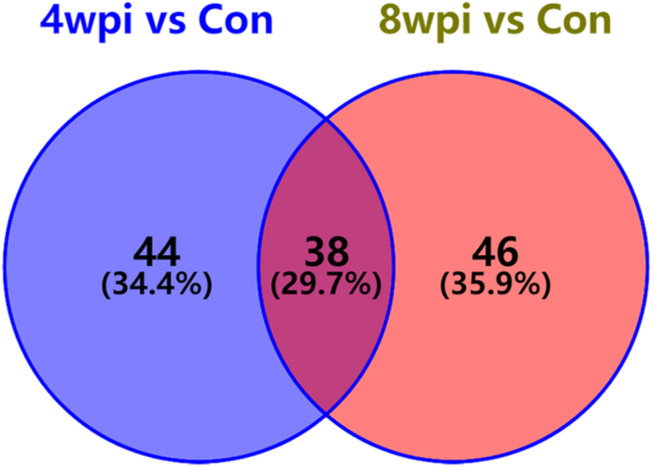
Venn diagram displaying the common and unique metabolites among the two infection groups vs control group. There were 38 significantly differential metabolites were shared in two infected groups. 4 wpi vs con, 4 wpi group vs control group; 8 wpi vs con, 8 wpi group vs control group.

According to the *P*-value <0.05 and VIP >1, a total of 396 and 242 potential metabolites of spleen involved in clonorchiasis were screened out in ESI+ and ESI− modes, respectively (Supplementary Table S2). And the results showed a clear difference between different groups in the volcano plots (Figure 3 and Supplementary Figure S4) and heatmaps (Figure 4 and Supplementary Figure S5). To obtain the significantly differential metabolites, based on FC > 2 or <0.5, and VIP > 1.5, we further identified 82 and 84 metabolites in ESI+ and ESI− modes, respectively. Next, a Venn diagram was constructed to show the metabolites in different infection stages (Figure 5). There were 38 significantly different metabolites shared in the two infected groups compared with the control group. These metabolites included amino acids, nucleotides, carboxylic acids, lipids and carbohydrates. Notably, significant differences were observed in the amino acid profiles.

**FIGURE 6:**
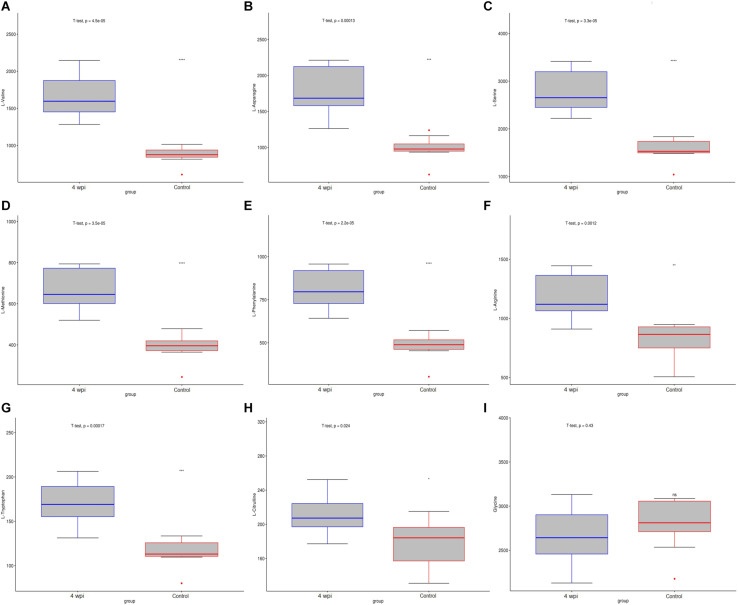
The distribution of amino acids were present by boxplot between 4 wpi vs control group. **(A)** L-Valine; **(B)** L-Asparagine; **(C)** L-Serine; **(D)** L-Methionine; **(E)** L-Phenylalanine; **(F)** L-Arginine; **(G)** L-Tryptophan; **(H)** L-Citrulline; **(I)** Glycine. Boxes represent the interquartile ranges (IQRs) between the first and third quartiles, and the line inside the box represents the median. The amino acid content was compared with the median value between the two groups. Circles represent outliers. *P < 0.05, ***P < 0.001.

Based on untargeted profiling analysis, targeted UHPLC-MRM-MS/MS quantitative analysis of 25 key amino acids was established and further evaluated in the spleen. Among them, 15 amino acids screened by untargeted profiling can be accurately quantified and mostly identified increased during clonrochiasis, including L-valine, L-asparagine, L-serine, L-methionine, L-phenylalanine, L-histidine, L-threonine, L-tryptophan, L-arginine, L-tyrosine, L-proline, L-alanine, and L-glutamine, expect for L-citrulline decreased at 8 wpi. While glycine was identified decreased during clonrochiasis. Several amino acids are represented using boxplots in Figure 6 and Supplementary Figure S6.

### Metabolic Pathways Affected by *C. sinensis* Infection

**FIGURE 7:**
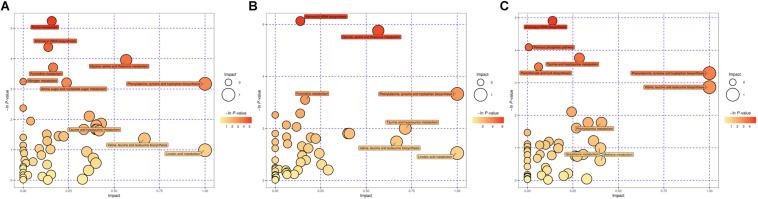
The pathway analysis during *C. sinensis* infection in ESI+ mode. Plots depict the pathway impacts of the key metabolites (*x*-axis) and the computed metabolic pathway as a function of –log (P) (*y*-axis) that different between the 4 wpi vs control **(A)**, 8 wpi vs control **(B)**, 8 vs 4 wpi **(C)**.

**FIGURE 8:**
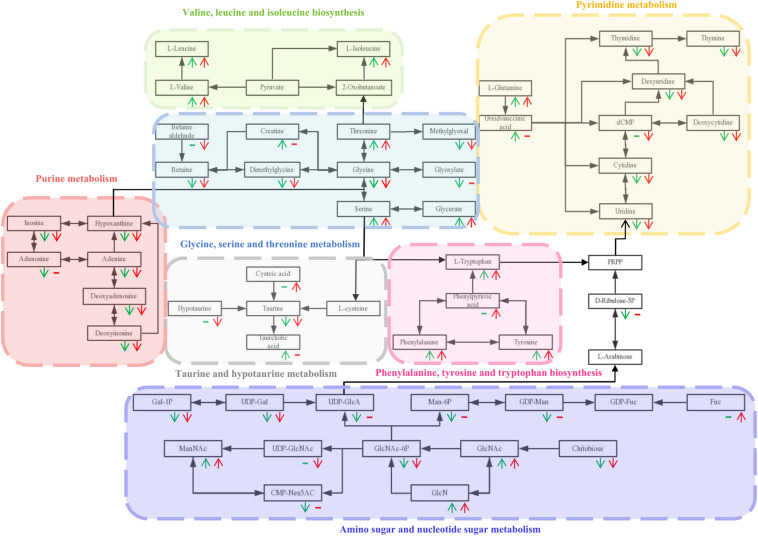
The integrated metabolic pathway shared in 4 and 8 wpi during *C. sinensis* infection. Beneath each metabolite, green and red represent 4 and 8 wpi compared with the control, respectively. The arrows indicate up and down-regulation of metabolites, respectively. Horizontal lines indicate that the metabolites are not significantly different in this infection stage.

Based on the *P*-value (*P*-value < 0.05) and pathway impact value, significant differential metabolic pathways involved in clonorchiasis were estimated by KEGG annotation and MetaboAnalyst. As showed in the bubble chart, the differential metabolites were enriched in purine metabolism, aminoacyl-tRNA biosynthesis, pyrimidine metabolism, phenylalanine, tyrosine and tryptophan biosynthesis at 4 wpi; and aminoacyl-tRNA biosynthesis, pyrimidine metabolism, purine metabolism, phenylalanine, tyrosine and tryptophan biosynthesis at 8 wpi (Figure 7 and Supplementary Figure S7). As shown in the Figure 8 and Supplementary Tables S3, S4, purine metabolism, pyrimidine metabolism, glycine, serine and threonine metabolism, and phenylalanine, tyrosine and tryptophan biosynthesis were integrated analyzed in the network.

## Disscusion

Clonorchiasis is a public health problem worldwide with epidemiological significance (Lun et al., 2005). However, the underlying mechanism of clonorchiasis is not fully clearly. Recently, some studies have evaluated endogenous metabolites and identified perturbed metabolic pathways in parasitic diseases (Besteiro et al., 2010; Notarangelo et al., 2014). However, it is unknown whether *C. sinensis* infection could result in the imbalance of spleen metabolism and related biochemical pathways. In this study, the untargeted and targeted metabolomic methods were carried out to analyze the spleen metabolic profiles and related metabolic pathways in clonorchiasis. These results may help to explore the pathophysiology mechanism, making diagnosis and prevention strategies for clonorchiasis.

A number of animal models have been used to investigate the interaction of host and *C. sinensis*, such as rabbits, mice, hamsters, and rats. With low recovery rates and underdevelopment of worms (the parameter for host susceptibility), mice are not suitable hosts of *C. sinensis* regardless of its strain (Uddin et al., 2012). Because of the limited commercially available antibodies or probes, it is not appropriate for evaluating the pathogenesis or immune responses in hamsters (Uddin et al., 2012). Although the rats develop resistance to reinfection by *C. sinensis* (Quan et al., 2005), with the advantages of small size, low cost, wide source, easy feeding, and suitable for large-scale observation, rats have been broadly used as animal models for exploring pathogenesis of clonrochiasis (Choi et al., 2004; Quan et al., 2005; Sohn et al., 2006; Han et al., 2016, 2017). In addition, we could get better adults recovery rates and development of worms in rats models. Some studies also had used rats models to investigate the immunity of clonorchiasis (Zhou et al., 2013; He et al., 2014). Thus, the rat models are considered as useful models for analysis the metabolomics of *C. sinensis* infection, and has more reference value for human body. First, it is difficult to collect the samples from clonorchiasis patients, including liver and spleen tissues. Second, some functions of the spleen from animal models are similar with human body. After preliminary studies in rat models, some metabolites are obtained and then could be further verified in clonrochiasis patients. Therefore, it is suited for making rat models for metabolomics analysis of clonorchiasis.

As a major immune organ, the spleen plays a crucial role in innate and adaptive immune responses (Zhao et al., 2015). Innate immunity is important for the immune surveillance of inner and outer threats as well as initial host defense responses (Chen J. et al., 2017). Immune surveillance could protect the host against parasites. Dendritic cells (DCs) have an essential role in immune surveillance (Cabeza-Cabrerizo et al., 2019). In addition, Toll-like receptors (TLRs) are important for against microbial infection (Pulendran et al., 2010). During *C. sinensis* infection, DCs played a key role in immune surveillance through TLR-mediated pathway, with increased levels of IFN-γ, IL-6, TNF-α, and IL-10 in the splenocytes (Hua et al., 2018). In view of the importance of the spleen in immune surveillance during *C. sinensis* infection, we chose the spleen as the research object.

In this study, a total of 396 and 242 significant differential metabolites were identified in ESI+ and ESI− modes, respectively. These metabolites covered amino acids, nucleotides, carboxylic acids, lipids and carbohydrates. The results suggested that *C. sinensis* infection could induce systemic metabolic perturbations in the spleen. In addition, there were 38 significantly different metabolites shared in the two infected groups compared with the control group, according to FC > 2 or <0.5, and VIP > 1.5. These metabolites might be a direct signal of *C. sinensis* activity or the consequence of the host response to the parasite. Generally, these dynamic metabolites not only could suggest the interaction between the host and *C. sinensis*, but also might perturb the biochemical profiles of them (Abdelrazig et al., 2017).

Our results found that *C. sinensis* infection induced significant changes in amino acids. In addition, 15 amino acids screened by untargeted profiling can be accurately quantified using targeted analysis during clonrochiasis. These results further corroborate the difference in abundance of amino acids between infection and control groups. Similarly, dysregulation in levels of amino acids metabolism were found and involved in toxoplasmosis and schistosomiasis (Wu et al., 2010; Chen et al., 2018). These amino acids not only affect cell signaling, recruitment and proliferation during infection, but reflect an immune response to infection and/or tissue injury and repair (Chandler et al., 2016). Besides, amino acids are also given the functions, such as energy dissipation, synthesis of basic organic molecules of proteins which could protect the host’s innate immune response (Li P. et al., 2009; Lu et al., 2017). For example, L-leucine, L-glutamine, and L-valine have the same trend (up-regulation) at 4 and 8 wpi groups compared with the control, respectively. Moreover, compared with 4 wpi, these three animo acids were also found increased in 8 wpi. Leucine could provide energy when continuous energy consumption (Gironès et al., 2014). Glutamine is an important energy source for mitochondria and could be utilized by lymphocytes (Yaqoob and Calder, 1997). L-valine could alter the function of immune cells (Kakazu et al., 2007). Therefore, it indicates that the host is required to consume more energy, which is consistent with the results from *Schistosoma mansoni* infection (Li J. V. et al., 2009). Except that, we also found L-tryptophan, L-tyrosine, and L-arginine increased in *C. sinensis*-infected rats, compared with controls. These three amino acids were considered as essential amino acids required by the parasite to sustain its own growth (Silva et al., 2002; Marino and Boothroyd, 2017). Energy metabolism plays a key role in facilitating the adaptation of adult flukes to crowded habitat and hostile environment (Li et al., 2020). While, some metabolites were found fluctuated with disease progression. One possible interpretation of this result is that the host makes the regulation of the metabolism level. Therefore, the abnormality of these amino acids suggested that the host needs to maintain the energy requirements, sustain its own growth and regulates the immune system to interact with *C. sinensis* infection.

In addition, inosine and adenine were found down-regulated both in 4 and 8 wpi, compared with the control. Numerous reports have suggested that inosine could effectively inhibit pro-inflammatory cytokines such as IFN-γ, TNF-α, and IL-12 *in vitro* and *in vivo* (Haskó et al., 2000; Mabley et al., 2003). Moreover the purines play a major role in regulating inflammatory and immune responses during diseases and reducing inflammatory tissue damage (Iregui et al., 2016). Therefore, these results indicate that the host regulates inflammation and immune responses to decrease infection in clonorchiasis.

Besides, deoxyinosine is involved in the purine metabolism pathway, which could be substituted for glucose as an energy source (Chen et al., 2019). Meanwhile, deoxyinosine could be catabolized into hypoxanthine, regulating energy metabolism (Lee et al., 2018). Thus the decreased deoxyinosine and hypoxanthine in this present study may provide less energy and lead to compromised spleen cell development. Additionally, deoxyadenosine could dampen the function of immune cells by triggering the caspase-3-mediated death of macrophages (Carrera et al., 1990; Thammavongsa et al., 2013), which was down-regulated in this present study. As a precursor of DNA, thymidine was down-regulated, which indicating the degree of lymphocytes proliferation (Wang et al., 2015). Taken together, the metabolites suppressed immunity was significantly decreased, indicating that the host actively regulates the immune response, despite the spleen cells were damaged and the proliferation was inactive.

**FIGURE 9:**
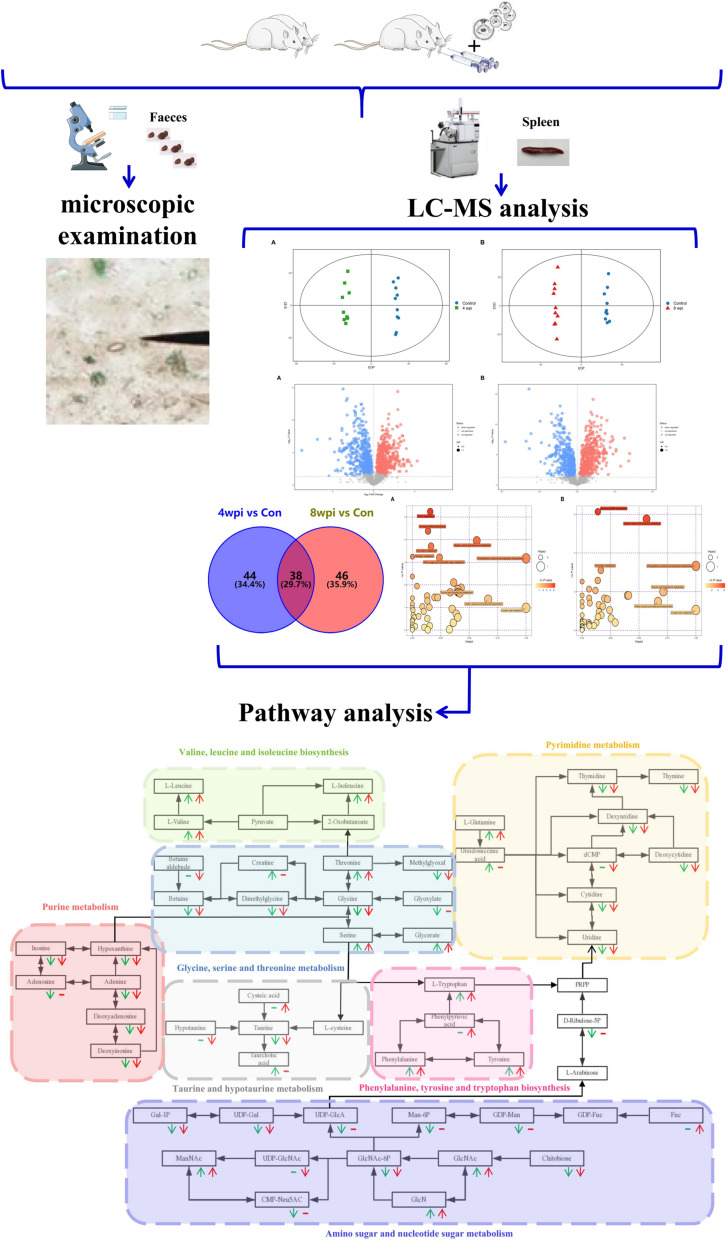
Flow chart of metabolomics in spleen of rats infected with *Clonorchis sinensis.*

Generally, our research established the *C. sinensis*-infected rats model to anlayze the spleen metabolomics for the first time, revealing the biochemical characteristics and molecular mechanisms of infection (Figure 9). However, the present study also has several limitations. First, the numbers of the samples were low. Thus, larger samples with time-matched control groups should be assessed in future studies. Second, owing to the complex and dynamic cellular heterogeneity of the spleen, the altered metabolite may be the result of differential expression of spleen cell. However, our research serves to deepen the understanding of the molecular mechanisms of clonorchiasis. In the future, an individual cell population in the spleen will be selected for metabolomics or multi-omics joint analysis, and these differential metabolites requires further investigation.

## Conclusion

The non-targeted UHPLC-QTOF MS method was used to explore the metabolic profiles in the spleen of *C. sinensis*-infected rats in this study. Differential metabolites included amino acids (L-leucine, L-glutamine, and L-valine) and nucleotides (inosine, deoxyadenosine, thymidine, and deoxyinosine). In addition, a total of 15 amino acids screened by untargeted profiling were accurately quantified and identifed by the targeted UHPLC-MRM-MS/MS approach. Several metabolic pathways were associated with the pathogenesis of clonorchiasis, including aminoacyl-tRNA biosynthesis, pyrimidine metabolism, and phenylalanine, tyrosine, and tryptophan biosynthesis. These results could be contributed to understanding the immunoregulatory process of *C. sinensis* infection and providing new insights into the molecular mechanisms of host-parasite interactions.

## Data Availability Statement

All datasets presented in this study are included in the article/Supplementary Material.

## Ethics Statement

The animal study was reviewed and approved by the Medical Ethics Review Committee of Harbin Medical University.

## Author Contributions

SH and XZ conceived and designed the experiments. SH, XH, and XZ performed the experiments. SH and XH analyzed the data. SH, XH, RC, BS, YG, SD, and LL contributed to reagents, materials, and analysis tools. SH, XH, and XZ wrote the manuscript. All authors edited the manuscript, read, and approved the final version of the manuscript.

## Conflict of Interest

The authors declare that the research was conducted in the absence of any commercial or financial relationships that could be construed as a potential conflict of interest.

## Footnotes

1 http://mev.tm4.org/

2 www.genome.jp/kegg/

3 http://geneontology.org/

4 http://www.MetaboAnalyst.ca/

## Supplementary Material

Supplementary Figure S1 Principal component analysis (PCA) score scatter plots of splenic metabolites during *C. sinensis* infection. **(A)** PCA score scatter plot of splenic metabolites in the positive ion mode (ESI+); **(B)** PCA score scatter plot of splenic metabolites in the negative ion mode (ESI−); Control, healthy control; 4 wpi, 4 weeks post infection; 8 wpi, 8 weeks post infection; QC, quality control.

Supplementary Figure S2 Orthogonal partial least squares discriminant analysis (OPLS-DA) score scatter plots of splenic metabolites during *C. sinensis* infection in ESI− mode. **(A)** OPLS-DA score scatter plot of 4 wpi vs control in ESI− mode; **(B)** OPLS-DA score scatter plot of 8 wpi vs control in ESI− mode; **(C)** OPLS-DA score scatter plot of 8 vs 4 wpi in ESI− mode. Control, healthy control; 4 wpi, 4 weeks post infection; 8 wpi, 8 weeks post infection.

Supplementary Figure S3 Permutation test of OPLS-DA model showing the stability of the model in ESI− mode. **(A)** Permutation test of OPLS-DA model of 4 wpi vs control in ESI− mode; **(B)** Permutation test of OPLS-DA model of 8 wpi vs control in ESI− mode; **(C)** Permutation test of OPLS-DA model of 8 vs 4 wpi in ESI− mode; The abscissa indicates the displacement retention of the permutation test, and the ordinate indicates the value of R2Y or Q2. The green dot indicates the R2Y value obtained by the displacement test, the blue square indicates the Q2 value obtained by the permutation test, and the two dotted lines indicate the regression lines of R2Y and Q2, respectively. The point where the displacement retention is 1 is R2Y and Q2 of the original model.

Supplementary Figure S4 Volcano plot representation of the differential metabolites identified in ESI− mode. **(A)** 4 wpi vs control; **(B)** 8 wpi vs control; **(C)** 8 vs 4 wpi; Each point in the map represents a metabolite. The size of the scatter represents the VIP value of the OPLS-DA model, and the larger the scatter, the larger the VIP value. Scatter color represents the final screening result, red represents significant up-regulation, blue represents significant down-regulation, and gray represents non-significant difference metabolites.

Supplementary Figure S5 Heatmaps representation of the differential metabolites identified between different infection group vs control group in ESI− mode. **(A)** 4 wpi vs control; **(B)** 8 wpi vs control; **(C)** 8 vs 4 wpi; The color blocks at different positions represent the relative expression of metabolites at corresponding positions, red represents up-regulated, and blue represents down-regulated.

Supplementary Figure S6 The distribution of amino acids were present by boxplot between 8 wpi vs control group. **(A)** L-Valine; **(B)** L-Asparagine; **(C)** L-Serine; **(D)** L-Methionine; **(E)** L-Phenylalanine; **(F)** L-Arginine; **(G)** L-Tryptophan; **(H)** L-Citrulline; **(I)** Glycine. Boxes represent the interquartile ranges (IQRs) between the first and third quartiles, and the line inside the box represents the median. The amino acid content was compared with the median value between the two groups. Circles represent outliers. ^∗^*P* < 0.05, ^∗∗∗^*P* < 0.001.

Supplementary Figure S7 The pathway analysis during *C. sinensis* infection in ESI− mode. Plots depict the pathway impacts of the key metabolites (*x*-axis) and the computed metabolic pathway as a function of **−**log (P) (*y*-axis) that different betweenthe 4 wpi vs control **(A)**, 8 wpi vs control **(B)**, 8 vs 4 wpi **(C)**.

Supplementary Table S1 Parameters of PCA and OPLS-DA models based on the date from sub-comparisons in the positive ion mode (ESI+) and the negative ion mode (ESI−).

Supplementary Table S2 List of metabolites identified in the entire infection process.

Supplementary Table S3 Summary of the top 10 pathway analysis using MetaboAnalyst during different infection groups in ESI+ mode.

Supplementary Table S4 Summary of the top 10 pathway analysis using MetaboAnalyst during different infection groups in ESI− mode.
