# Supplementary figures and images for: Perturbations of Metabolomic Profiling of Spleen From Rats Infected With Clonorchis sinensis Determined by LC-MS/MS Method
Source: Front Mol Biosci. 2020 Oct 6;7:561641. doi: 10.3389/fmolb.2020.561641 (PMC7574862; doi:10.3389/fmolb.2020.561641)

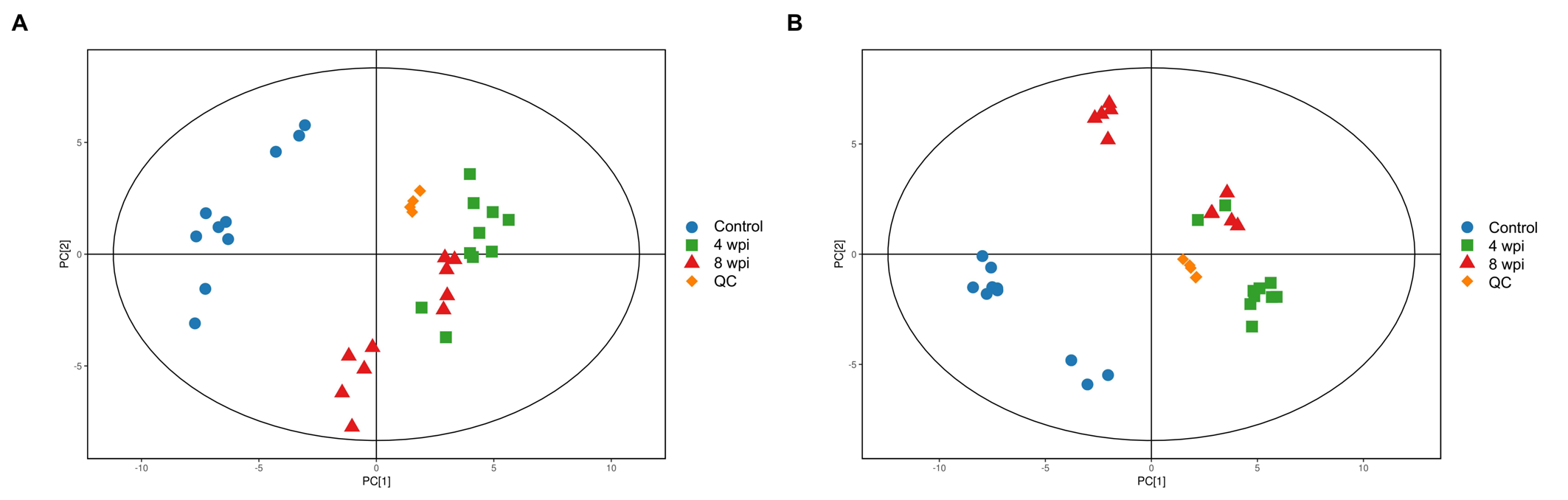

Supplement: Supplementary Figure S1 — Principal component analysis (PCA) score scatter plots of splenic metabolites during C. sinensis infection. (A) PCA score scatter plot of splenic metabolites in the positive ion mode (ESI+); (B) PCA score scatter plot of splenic metabolites in the negative ion mode (ESI−); Control, healthy control; 4 wpi, 4 weeks post infection; 8 wpi, 8 weeks post infection; QC, quality control. [file Image_1.TIF]

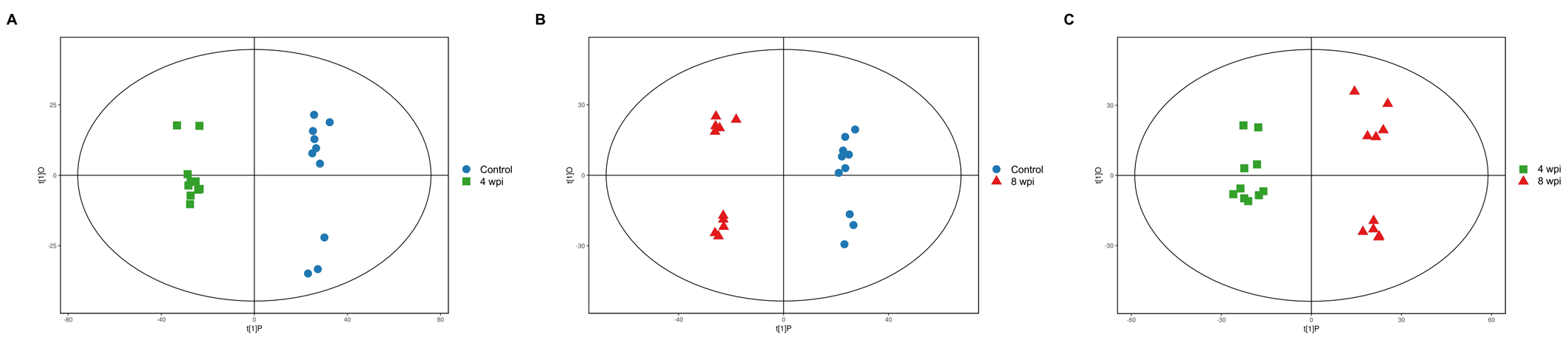

Supplement: Supplementary Figure S2 — Orthogonal partial least squares discriminant analysis (OPLS-DA) score scatter plots of splenic metabolites during C. sinensis infection in ESI− mode. (A) OPLS-DA score scatter plot of 4 wpi vs control in ESI− mode; (B) OPLS-DA score scatter plot of 8 wpi vs control in ESI− mode; (C) OPLS-DA score scatter plot of 8 vs 4 wpi in ESI− mode. Control, healthy control; 4 wpi, 4 weeks post infection; 8 wpi, 8 weeks post infection. [file Image_2.TIF]

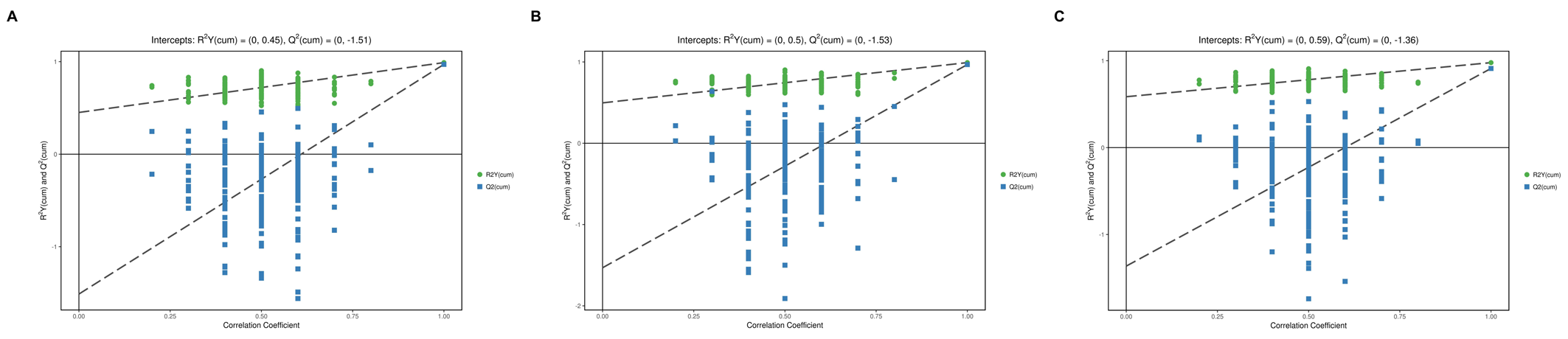

Supplement: Supplementary Figure S3 — Permutation test of OPLS-DA model showing the stability of the model in ESI− mode. (A) Permutation test of OPLS-DA model of 4 wpi vs control in ESI− mode; (B) Permutation test of OPLS-DA model of 8 wpi vs control in ESI− mode; (C) Permutation test of OPLS-DA model of 8 vs 4 wpi in ESI− mode; The abscissa indicates the displacement retention of the permutation test, and the ordinate indicates the value of R2Y or Q2. The green dot indicates the R2Y value obtained by the displacement test, the blue square indicates the Q2 value obtained by the permutation test, and the two dotted lines indicate the regression lines of R2Y and Q2, respectively. The point where the displacement retention is 1 is R2Y and Q2 of the original model. [file Image_3.TIF]

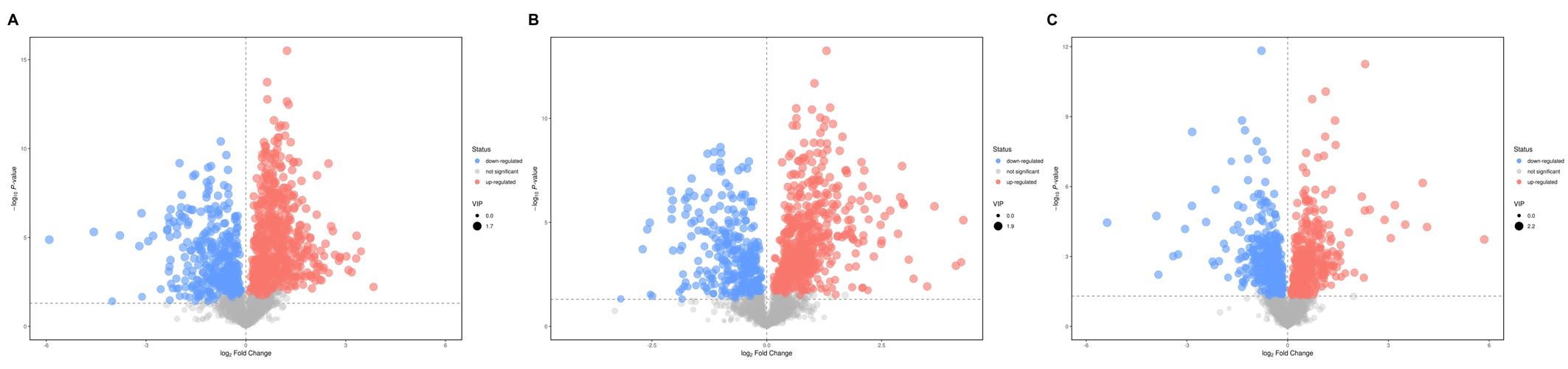

Supplement: Supplementary Figure S4 — Volcano plot representation of the differential metabolites identified in ESI− mode. (A) 4 wpi vs control; (B) 8 wpi vs control; (C) 8 vs 4 wpi; Each point in the map represents a metabolite. The size of the scatter represents the VIP value of the OPLS-DA model, and the larger the scatter, the larger the VIP value. Scatter color represents the final screening result, red represents significant up-regulation, blue represents significant down-regulation, and gray represents non-significant difference metabolites. [file Image_4.TIF]

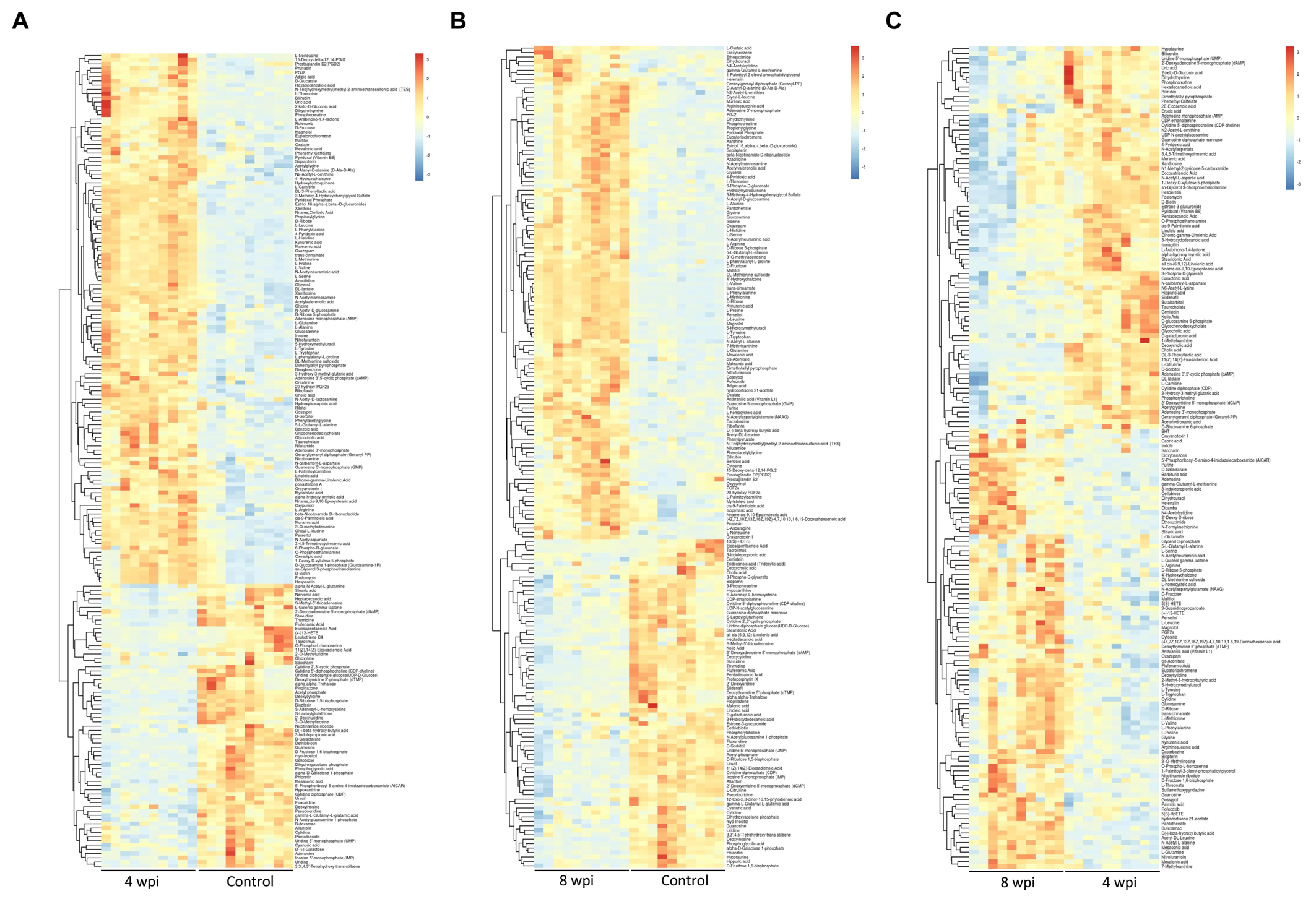

Supplement: Supplementary Figure S5 — Heatmaps representation of the differential metabolites identified between different infection group vs control group in ESI− mode. (A) 4 wpi vs control; (B) 8 wpi vs control; (C) 8 vs 4 wpi; The color blocks at different positions represent the relative expression of metabolites at corresponding positions, red represents up-regulated, and blue represents down-regulated. [file Image_5.TIF]

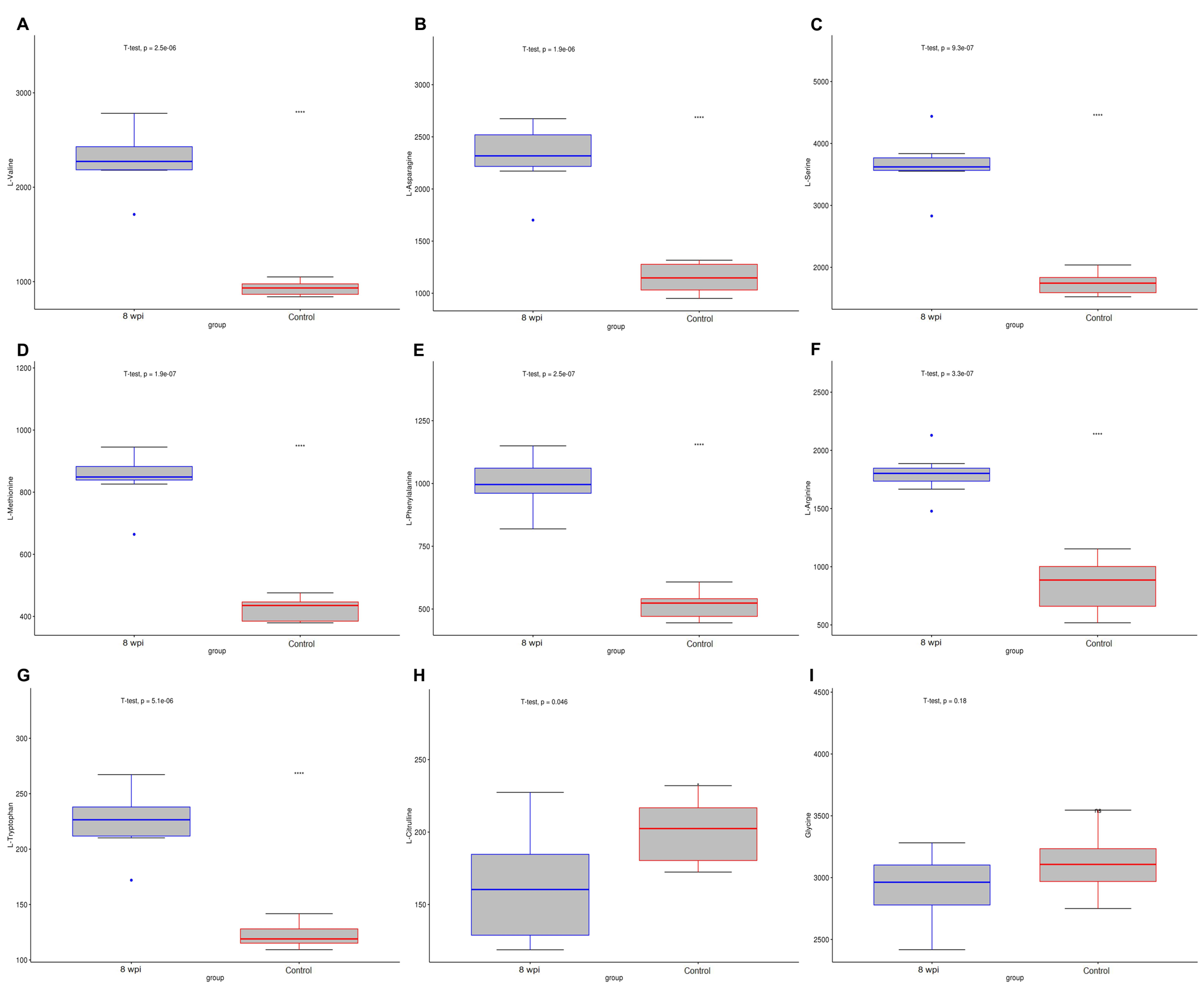

Supplement: Supplementary Figure S6 — The distribution of amino acids were present by boxplot between 8 wpi vs control group. (A) L-Valine; (B) L-Asparagine; (C) L-Serine; (D) L-Methionine; (E) L-Phenylalanine; (F) L-Arginine; (G) L-Tryptophan; (H) L-Citrulline; (I) Glycine. Boxes represent the interquartile ranges (IQRs) between the first and third quartiles, and the line inside the box represents the median. The amino acid content was compared with the median value between the two groups. Circles represent outliers. ∗P < 0.05, ∗∗∗P < 0.001. [file Image_6.TIF]

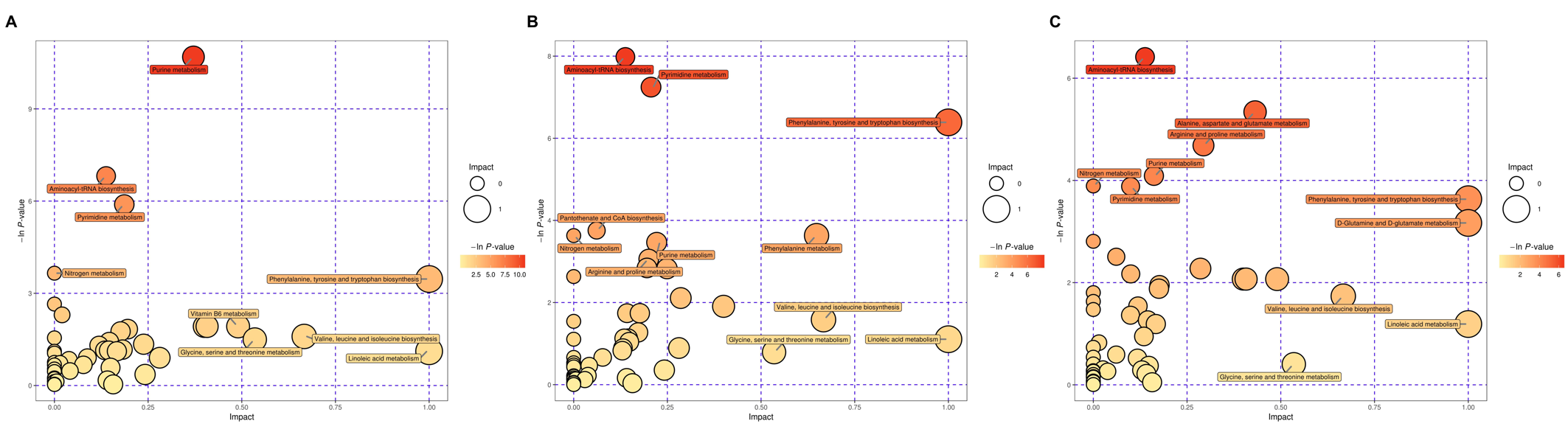

Supplement: Supplementary Figure S7 — The pathway analysis during C. sinensis infection in ESI− mode. Plots depict the pathway impacts of the key metabolites (x-axis) and the computed metabolic pathway as a function of −log (P) (y-axis) that different betweenthe 4 wpi vs control (A), 8 wpi vs control (B), 8 vs 4 wpi (C). [file Image_7.TIF]
